# Indoor Spatial Updating with Reduced Visual Information

**DOI:** 10.1371/journal.pone.0150708

**Published:** 2016-03-04

**Authors:** Gordon E. Legge, Rachel Gage, Yihwa Baek, Tiana M. Bochsler

**Affiliations:** Department of Psychology, University of Minnesota, Twin Cities, Minnesota, United States of America; University of Waterloo, CANADA

## Abstract

**Purpose:**

Spatial updating refers to the ability to keep track of position and orientation while moving through an environment. People with impaired vision may be less accurate in spatial updating with adverse consequences for indoor navigation. In this study, we asked how artificial restrictions on visual acuity and field size affect spatial updating, and also judgments of the size of rooms.

**Methods:**

Normally sighted young adults were tested with artificial restriction of acuity in Mild Blur (Snellen 20/135) and Severe Blur (Snellen 20/900) conditions, and a Narrow Field (8°) condition. The subjects estimated the dimensions of seven rectangular rooms with and without these visual restrictions. They were also guided along three-segment paths in the rooms. At the end of each path, they were asked to estimate the distance and direction to the starting location. In Experiment 1, the subjects walked along the path. In Experiment 2, they were pushed in a wheelchair to determine if reduced proprioceptive input would result in poorer spatial updating.

**Results:**

With unrestricted vision, mean Weber fractions for room-size estimates were near 20%. Severe Blur but not Mild Blur yielded larger errors in room-size judgments. The Narrow Field was associated with increased error, but less than with Severe Blur. There was no effect of visual restriction on estimates of distance back to the starting location, and only Severe Blur yielded larger errors in the direction estimates. Contrary to expectation, the wheelchair subjects did not exhibit poorer updating performance than the walking subjects, nor did they show greater dependence on visual condition.

**Discussion:**

If our results generalize to people with low vision, severe deficits in acuity or field will adversely affect the ability to judge the size of indoor spaces, but updating of position and orientation may be less affected by visual impairment.

## Introduction

Spatial updating refers to the ability to keep track of one’s position and orientation while moving through an environment [1]. Spatial updating is important for keeping track of one’s current position with respect to key locations such as the starting point. In indoor environments, the ability to judge the size and shape of a space is also important for safe mobility and navigation. People with impaired vision from eye disease may be less accurate in making these judgments with adverse consequences for indoor navigation. In this study, we asked how artificially reduced restrictions on visual acuity and field size affect spatial updating, and also judgments of the size and shape of rooms. Experiments with normally sighted subjects and artificial visual restrictions permit control over stimulus attributes, and provide a baseline for future studies with visually impaired subjects.

This investigation is part of a multi-disciplinary project—called Designing Visually Accessible Spaces—focused on understanding and enhancing visual accessibility for people with impaired vision. Visual accessibility is the use of vision to travel efficiently and safely through an environment, to perceive the spatial layout of key features in the environment, and to keep track of one’s location in the environment. In previous psychophysical work, we focused on factors affecting the visibility of local features present in indoor spaces, such as steps, ramps, and geometrically simple convex objects [2–5]. These studies involved both normally sighted subjects with artificial acuity reduction and low vision subjects. We investigated the effects of acuity, viewing distance, lighting arrangement and target contrast on the visibility of these objects.

In the current study, we have focused on global features of indoor spaces. We asked how different forms of reduced visual information affect spatial updating. Normally sighted subjects wore goggles to simulate reduced acuity or severe field restriction. In a separate paper, we will compare these findings with results from a group of subjects with low vision performing the same tasks. This second paper will also report on spatial updating without visual cues by blind-folded subjects with normal vision and low vision, and also blind subjects.

The effects of blur and field restriction will depend on the cues available for spatial updating and judging room dimensions. For example, blur is likely to reduce the usefulness of some cues for distance such as familiar size and texture gradients conveyed by high spatial frequencies. Field restriction may affect the ability to perceive large features conveying room shape, or to use optic flow cues for spatial updating. In the Discussion section, we will interpret our empirical results in the context of the cues available with visual restrictions.

Our subjects were tested in unfamiliar rooms in a large office building. In the main experiment, subjects walked along a three-segment path in the room. At the end of the path, they were asked to make spatial-updating judgments, consisting of estimates of the distance and direction to the starting location of the path, and also distance and direction to a target object (a bean bag dropped at the first way point). The subjects were also asked to estimate the length and width of the rectangular rooms. All of these judgments were made without looking back at the starting location or target, and with the visual restriction in place.

The visual restrictions included mild and severe blur, and field restriction. Previous studies have shown that moderately severe blur (in the range 20/500 to 20/800) has little impact on the perceived distance of visible objects [6, 7], but does affect learning of spatial layouts [8, 9]. In our study, subjects wore blurring goggles that artificially reduced acuity to Snellen 20/135 (Mild Blur) and 20/900 (Severe blur). Creem-Regehr et al. [10] found that restriction of the field of view to 42° horizontal by 32° vertical had no effect on judgments of absolute distance to targets over a range of 2 to 12 m. Peruch et al. [11] performed a spatial updating task in virtual reality using purely visual stimuli and found no effect of the field of view from 40° to 80°. But Fortenbaugh, Hicks and Turano [12] observed distortions in the memory representations of the layout of landmarks by a group of subjects with restricted visual fields from retinitis pigmentosa; greater errors were associated with narrower fields. In an obstacle avoidance task with parametric variation of field size, Hassan et al. [13] found performance deficits with restricted fields from roughly 10° to 30° for low, medium and high-contrast conditions. In our Narrow Field condition, subjects were restricted to a field of view subtending 8°.

We included updating with respect to a target within the space, in addition to the starting location, because previous work has suggested that people remember geometrical properties of a space (like the doorway starting point) better than objects within the space [14]. We asked whether subjects’ estimates of distance and direction to the starting location would be more accurate than those to the target.

We also included a manipulation termed the “Preview” condition. In this case, subjects viewed the space from the doorway for 10 seconds without any visual restriction. They were then guided along the three-segment path blindfolded, followed by their updating and room-size judgments. The preview condition was included to determine whether visual imagery, gleaned during the preview, and presumably encoded in visual working memory, would facilitate spatial updating. Previous studies have indicated that visual preview of a space can sometimes enhance the precision of nonvisual spatial updating [15, 16].

Loomis et al. [1] propose that there are two distinct, but interrelated, methods for spatial updating, termed piloting and path integration. Piloting relies on reference to external visual or auditory landmarks for spatial updating. In contrast, path integration depends on proprioceptive, vestibular, and optic or acoustic flow information about self-motion for updating and might be less dependent on visual input. If subjects used external landmarks (piloting) for spatial updating, we anticipated that restricting visual input would make their judgments less accurate.

But vision is not necessary for path integration; it can be accomplished blind [1]. Vestibular and proprioceptive cues during movement can be used for spatial updating and may even take precedence over vision under certain conditions [17, 18]. Vestibular and proprioceptive information may be especially critical for blind and low-vision pedestrians, who navigate with limited or no visual input.

If subjects rely exclusively on proprioceptive and vestibular cues in path integration, visual restriction would not affect spatial-updating performance. To evaluate the impact of reducing proprioceptive cues, we conducted a second experiment in which subjects were pushed in a wheelchair along the three-segment path, rather than walking, prior to making their spatial updating judgments.

To summarize, we asked how artificial acuity and field restrictions affected access to two important types of global information relevant to indoor navigation—information about one’s current position and orientation, and information about room size and shape. We also asked if reduced proprioceptive information affects spatial updating with visual restrictions.

## Methods

### Participants

In Experiment 1, subjects were tested after walking, and in Experiment 2, subjects were tested after being pushed in a wheelchair. 32 normally sighted students at the University of Minnesota participated in Experiment 1 and 16 in Experiment 2. Mean acuities (Lighthouse Distance Visual Acuity chart) were 20/15.9 (Exp. 1) and 20/15.2 (Exp. 2). Mean contrast sensitivities (Pelli-Robson chart) were 1.98 (Exp. 1) and 2.0 (Exp. 2). Each subject completed the experiment in one session lasting one to two hours. The experimenter obtained written informed consent with procedures approved by the University of Minnesota’s IRB.

### Experiment 1: Walking

#### Test spaces and paths

**Fig 1:**
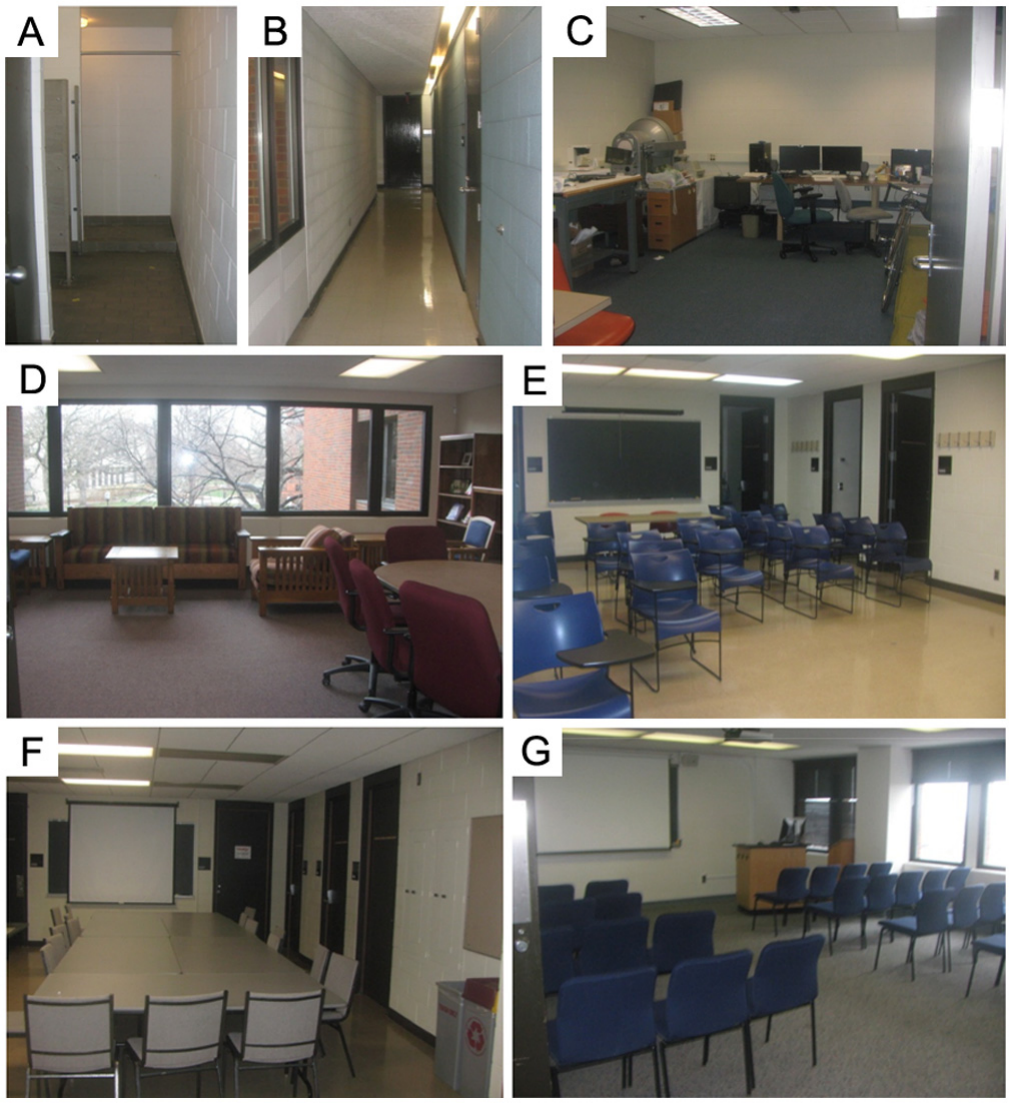
Photos from the seven rectangular test spaces. The dimensions of the seven spaces were (Door side × Non-door side in ft) A: 7.6 × 15.2. B: 4.3 × 44.4. C: 16.2 × 20.0. D: 19.9 × 22.1. E: 32.7 × 16.6. F: 33.2 × 16.6. G: 27.1 × 23.7.

The experiment took place in seven different rectangular spaces (Fig 1) in a building on the University of Minnesota, Twin Cities campus. Six were rooms and the seventh was a hallway.

We chose spaces with a range of sizes and rectangular aspect ratios in order to explore the impact of room dimensions. The spaces were a mix of classroom, meeting, and office space, containing typical furniture. All the spaces had overhead fluorescent lights and three had windows admitting natural daylight. Three rooms had carpeted floors, and the other four had light-colored linoleum flooring.

**Fig 2:**
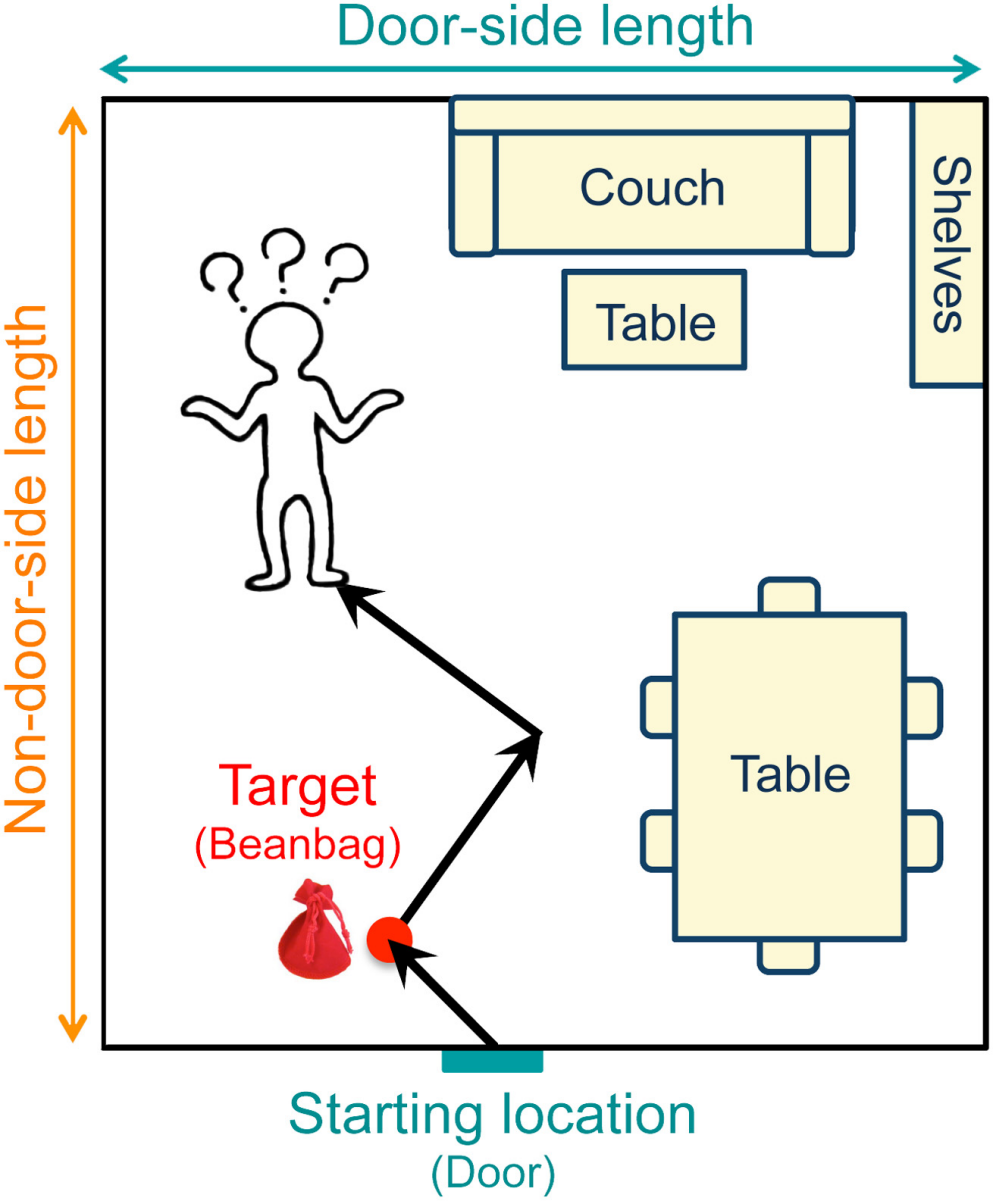
Schematic diagram illustrating the three-segment path. An experimenter guided the subject along a three-segment path beginning at the doorway. At the first waypoint, the subject dropped a beanbag, referred to as the target. At the end of the path, the subject made judgments about the distance and direction to the starting location and target, and also estimated the door side and non-door side dimensions of the space.

Different three-segment walking paths (Fig 2) were devised for each space. We tried to design the routes so that path length did not correlate with room size.

The experimenter led the subject along the path using a two-foot-long, half-inch diameter wooden rod. The experimenter held one end of the rod and the subject held the other end, keeping it flush with the hip so that the subject would accurately follow the path. Three tape marks on the floor of the space provided the experimenter with the position and orientation to line up the subject at the end of each segment. Path segments were approximately three, six, or nine feet long (range from 2 ft 10 inches to 8 ft 10 inches). None of the turning angles were 90°; most were between 20° and 60°, with a range from 3° to 69°, and included an equal number of clockwise and counterclockwise turns.

The subject dropped the target (beanbag) at the end of the first segment and made six spatial estimates at the end of the third segment in response to questions from the experimenter. See the description of Response Measures below.

#### Viewing conditions

Subjects were tested in six visual conditions—a control condition and five visual restriction conditions—and also two auditory conditions. Description of the two auditory conditions, in which subjects had no visual input, will be reported in a separate article, along with data for low-vision and blind subjects.

In the visual-restriction conditions, subjects wore noise-reducing earmuffs with earphones playing auditory white noise to mask acoustic cues. The volume was kept loud enough to mask most environmental noise, but at a level permitting voice communication with the experimenter.

The six visual conditions were:

1. Wide Field—Subjects used their normal, binocular vision, including any habitual corrections (glasses or contacts). They were not permitted to look back at the starting point or target location. This forced them to use spatial updating information gathered along the path, rather than relying on a visual-distance estimate at the end of the path. The experimenter monitored the subjects’ facing direction.
2. Narrow Field—The subject’s visual field was restricted to 8° using a cone mounted on goggles. Previous studies have shown that limited field of view influences mobility in people with low vision who are walking [19, 20]. Field restriction can also affect driving [21–23]. The field size of 8° was chosen because it is small enough to affect navigation, but large enough to permit access to some visual information.
3. Mild Blur—One Bangerter Occlusion Foil [24] was attached to one side of a clear acrylic lens and mounted in a welding goggle frame. Acuity through the monocular, blurring goggles was measured for each subject and averaged 20/135 on the Lighthouse Distance Acuity Chart.
4. Severe Blur—Two Bangerter foils were used, one on each side of an acrylic lens, in the goggles to blur vision to approximately 20/900.
  Viewing was monocular in the Mild Blur, Severe Blur and Narrow Field conditions, with the other eye occluded. Monocular viewing was used for practical reasons. It would have been difficult to match the foil orientations on two Bangerter filters. Also, it is difficult to simulate a narrow visual field binocularly due to the problem of binocular overlap. It has also been shown that judgments of absolute distance over the range from 2 to 12 m, roughly the range of room dimensions in our study, do not differ for monocular and binocular viewing [10].
5. Preview—The subject was permitted to view the space with free binocular viewing from the doorway for ten seconds. Vision was then occluded with a blindfold before the subject was guided along the 3-segment path.
6. Control—In the control trials, subjects returned to all of the test spaces and made estimates of the room length and width without any visual restrictions. Subjects were also guided along 3-segment paths in a series of five trials in one room, and asked to make spatial updating judgments with no visual constraints, that is, they were able to look back at the starting point and target location. The purpose of the Control conditions was to estimate baseline performance levels without any visual restrictions.

### Response Measures

After reaching the end of the three-segment path, the subject made six verbal responses, two concerning the room dimensions and four concerning spatial updating.

The subjects were asked to estimate the length and width of the space in feet or meters. In an effort to minimize ambiguity, we used the term “Door Side” to refer to the length of the side containing the entrance door, and “Non-Door Side” to refer to the orthogonal dimension.

When the room is viewed from the doorway, the Non-Door side estimate is an egocentric estimate of distance from oneself to the opposite wall, and the Door-Side estimate is an exocentric estimate of distance from the wall on the right to the wall on the left. We relied on two linear measures of room size (length and width in feet or meters) rather than one area measure in squared units for two reasons: 1) because we felt our subjects understood and were more comfortable with linear measures, and 2) we wanted to know whether performance would differ for the egocentric estimate (Non-Door Side) and exocentric estimate (Door Side.)

Our primary response measure was “*absolute error”* computed as the absolute difference between the physically measured length and the subject’s estimate. For instance if the physical length of the Door Side was 20 ft, and the subject reported either 22 or 18 ft, the absolute error was 2 ft.

There were four spatial-updating measures—distance and direction to the Start Location (the entrance door to the room), and the distance and direction to the Target (beanbag). Distances were estimated in the same way as the room dimensions, and corresponding absolute errors were calculated. The subjects understood that they were to estimate Euclidean distances to the Start and Target, not path length.

Subjects reported directions using a modified version of the four-quadrant verbal response measure introduced by Philbeck, Sargent, Arthur, and Dopkins [25]. They were instructed to imagine two axes running through their body, one from straight ahead to straight behind and one from left to right, defining four quadrants—Front Left, Front Right, Back Left and Back Right. The experimenter explained that reference directions straight ahead and straight behind were designated zero degrees and directions directly left and right were 90°. The subject reported direction by first indicating the quadrant and then the number of degrees away from the reference line. For example, the subject might say “the starting point is in the Back Left quadrant at 30°”. To make sure that subjects were not confusing left and right, or misspeaking, they were asked to confirm the intended quadrant with a hand gesture. Once again, our primary response measure was “*absolute error”* computed as the unsigned difference between the physically measured direction and the subject’s estimated direction.

#### Procedure

Prior to testing, subjects were familiarized with the distance and direction responses. They completed two practice trials: one with no visual or auditory restriction, and one with the blindfold and earmuffs. Successful completion of the practice trials confirmed that the subject understood the verbal reporting procedure, the nomenclature for room dimensions, and the path-following procedure.

During testing, an experimenter escorted the subject to the seven spaces. Before opening the door of a space to begin a trial, the experimenter ensured that the subject’s visual/auditory condition (earmuffs and/or simulated visual impairment) was in place. The subject began each trial at the doorway of the space, facing directly into the space, holding the rod in their right hand and the beanbag in their left hand. The experimenter always guided the subject along the route with the rod, even when the subject was participating in the Wide Field condition, and watched the subject to make sure they did not look back. When the experimenter and subject reach the end of the path, the subject remained facing forward with the visual/auditory condition in place while giving the six responses.

Subjects were exposed to one of the five visual-restriction or two auditory-restriction conditions in each of the seven rooms. For counterbalancing purposes, each subject was assigned to one of four groups (N = 8 per group). All members of a group did the same sequence of conditions in the same sequence of rooms. The four orders were chosen to ensure that each condition occurred in a variety of room sizes, so that room size was not confounded with condition.

The Control trials were conducted after completion of testing with the visual and auditory restrictions.

#### Data analysis

The counterbalancing meant that not all viewing conditions were tested in all rooms. In order to incorporate room size as a variable in our analysis, we used a linear mixed-effects (LME) model to fit the data using nlme package of R [26, 27]. The subjects were treated as random effects, and room size and viewing condition as fixed effects. The LME model can be expressed as:

$$y_{ij}=Α+Βx+V_{i}+E_{j}+e_{ij}$$ (1)

where y_ij_ is the model’s estimate of absolute error in ft for the jth subject in the ith condition, x is the physical value of the room dimension, A and B are mean values across all the subjects of the intercept and slope for a linear fit of the absolute errors in the Control condition, V_i_ is group mean additive error associated with the ith viewing condition, E_j_ is the random effect associated with the jth subject, and e_ij_ is the random error associated with observations by the jth subject in the ith condition. Interaction terms are not included in the equation because our primary interpretation of the results does not include interactions. The Results section does, however, include some brief comments on interactions.

For the room-size estimates, separate LME models were constructed for Door-Side and Non-Door-Side estimates. For the spatial updating measurements, the LME model revealed that the room-size effects were weak (see Results); as a consequence, we present the results without reliance on the LME model.

One subject was identified as an outlier in each of the two experiments, and data from both subjects were excluded from further analysis. These exclusions were based on a procedure within the nlme package to detect the data points with absolute standardized residuals that lie outside 99% CI of the standard normal distribution.

Significance of the fixed effects produced by the LME models were assessed using analysis of Variance (ANOVA) computed by the nlme package, giving F and P values for a Wald test [28].

### Experiment 2: Wheelchair

The same apparatus and procedure were used as in Exp. 1, with the following exceptions.

During experimental trials, the subject was seated in a wheelchair and pushed along the three-segment paths used in Exp. 1. The 38-inch-tall and 23-inch-wide transport chair (DanYang Maxthai Medical Equipment Co.) had handles behind the seat back so that it could be easily pushed. For each waypoint along the path, the experimenter positioned the chair so that the subject was seated directly above the tape marker in the correct orientation.

Two of the seven spaces were omitted (A and B in Fig 1) because the Door Side length was too narrow to maneuver the wheelchair along the paths. The logistics of testing were more time-consuming with the wheelchair. In order to complete testing in one session, the procedure was simplified in two ways. Only three of the five visual-restriction conditions were included: Wide Field, Narrow Field, and Severe Blur. Control measurements were obtained as in Exp. 1. The target manipulation (beanbag) was omitted.

## Results

### Experiment 1: Walking

#### Room dimensions

Subjects estimated the lengths of the Door Side and Non-Door Side dimensions of the test spaces.

We first consider the Control condition in which subjects estimated room dimensions with no visual restrictions. These estimates demonstrate the accuracy with which our normally sighted subjects could report the physical dimensions of the indoor spaces and provide a baseline for comparison with the visual-restriction conditions.

To determine whether subjects systematically overestimated or underestimated the room dimensions, we computed the ratios of subjects’ estimates to the physical lengths for the seven test spaces in the Control condition. Ratios greater than 1.0 represent overestimates of room size, and values less than 1.0 represent underestimates. F tests revealed that there was no significant effect of physical length on these ratios for either the Door Side or Non-Door Side.

The overall mean ratios were 1.08 for the Door Side, and 0.99 for the Non-Door Side. Mean ratios on a room-by-room basis ranged from 0.96 to 1.20 for the Door Side, and from 0.91 to 1.08 for the Non-Door Side.

These ratios indicate that subjects slightly overestimated the Door Side length (by about 8%), but exhibited no systematic over- or underestimate for the Non-Door Side length.

Our primary measure was Absolute Error, computed as the unsigned difference between the subject’s estimate in feet and the measured physical length.

**Fig 3:**
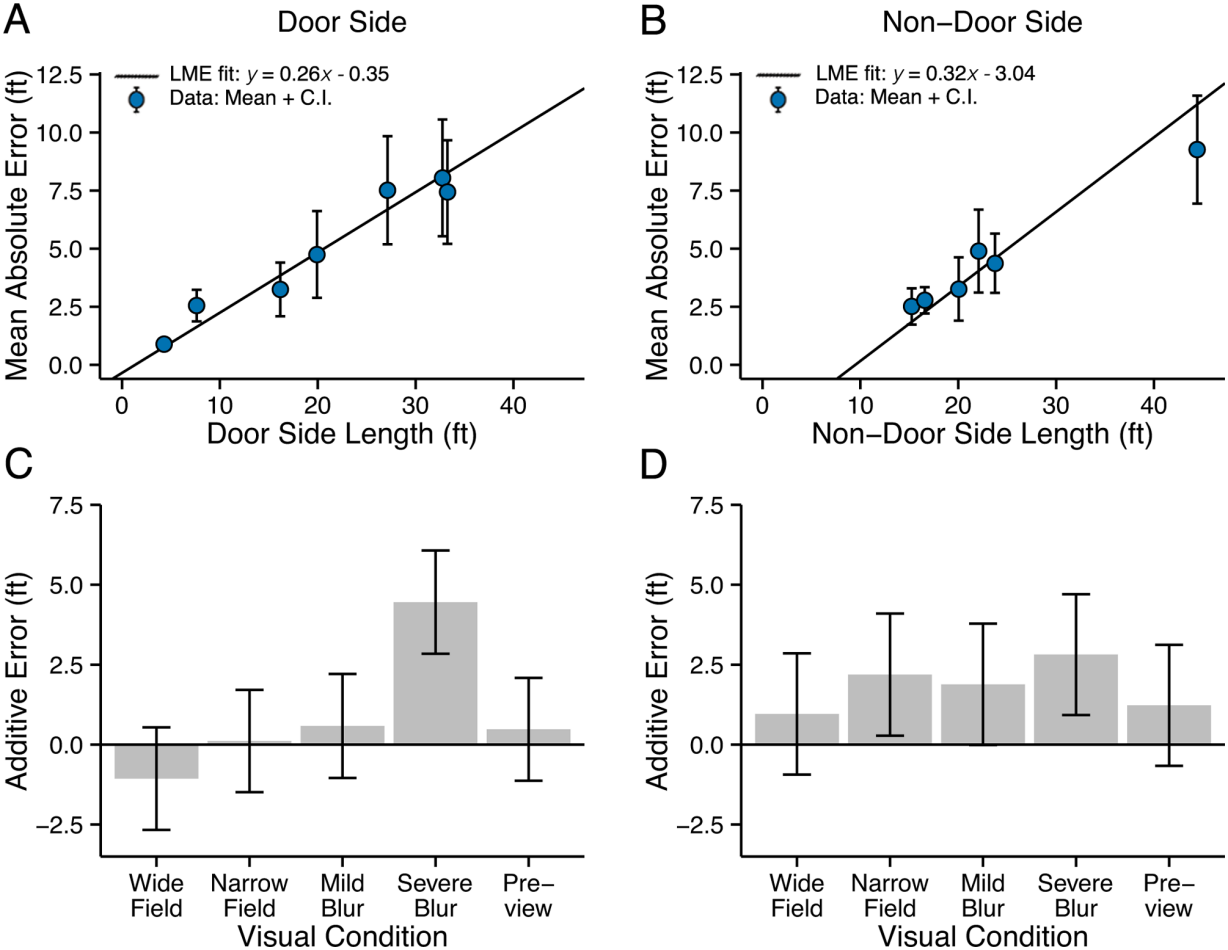
Experiment 1: Walking. Estimates of Room Dimensions. Mean values and 95% confidence intervals for the absolute error in the Control condition are plotted as a function of physical length for the Door Side (panel A) and Non-Door Side (Panel B). Straight lines show the LME fit. Panels C and D show the additive errors associated with the five viewing conditions, estimated from the LME model.

Panels A and B in Fig 3 plot mean absolute errors and their 95% confidence intervals as a function of the physical lengths of the Door Side and Non-Door Side of the seven test spaces for control data. The straight-line fit is from the LME model with the relevant room dimension as a fixed effect and viewing condition as a fixed effect. The rising line indicates that absolute error increased as physical length increased. The ratio of absolute error to physical length is a Weber fraction for room size estimation. For the Door Side, the overall mean Weber fraction was 0.26, with a range across rooms from 0.19 to 0.32. This means that our subjects averaged about 26% error in estimating Door-Side lengths, e.g., about 5.2 ft error for a length of 20 ft. For the Non-Door Side, the mean Weber fraction was 0.21 with a range from 0.16 to 0.28. This small difference in Weber fractions between the Door Side and Non-Door Side may not represent a reliable difference, given the noise in the data, and the unequal ranges and values of lengths tested for the two room dimensions.

A notable feature of the LME fit for the Non-Door Side (Panel B) is the substantially negative vertical intercept. We will comment on this below in connection with the impact of the range of room sizes on our results.

Panels C and D in Fig 3 show the additional absolute error associated with the other five visual conditions, relative to the Control condition (reference condition), based on the LME model (values of V_i_ in Eq 1 in the Method section). Since the Control condition is expected to represent the subjects’ best performance, poorer performance due to visual restriction would be represented by large positive values in panels C and D, representing the increase in absolute error relative to the Control condition. For the Door-Side (Panel C), Severe Blur was the only visual restriction showing a notable difference from the Control condition. For Severe Blur, the mean value of this additive term is 4.46 ft (95% CI: 3.65, 5.27 ft). To illustrate the meaning of this number, for a room with door side length of 20 ft, the LME model yields an absolute error with Severe Blur which is almost double the value in the Control condition (9.31 ft with Severe Blur and 4.85 ft in the Control condition). This larger absolute error with Severe Blur was associated with a tendency to overestimate the door side dimension; the mean ratio of estimated length to physical length for this condition was 1.15.

For the Non-Door Side (Panel D), relatively small but notable additional errors were associated with the Narrow Field condition (2.19 ft, CI = 1.91 ft), and Severe Blur (2.81 ft, CI = 1.89 ft). These values mean that for a room with Non-Door Side length of 20 ft, the LME model yields an absolute error of 3.36 ft in the Control condition, 5.55 ft with the Narrow Field, and 6.17 ft with Severe Blur.

Room size estimates in the Mild Blur condition were very similar to those in the Control conditions, as were the estimates in the Wide Field and Preview conditions. The restrictions associated with these conditions did not have an impact on subjects’ accuracy.

The ranges of room dimensions tested had two subtle effects on our data. For the seven test spaces, the Door-Side lengths varied from 4.3 ft to 33.2 ft, and the Non-Door Side lengths ranged from 15.2 ft to 44.4 ft. First, this difference may account for the negative vertical intercept in the LME fit for the Non-Door Side Control condition in Fig 3B. The intercept value of -3.04 ft is significantly different from 0. This value is physically impossible because absolute error is positive by definition. The presence of two smaller Door-Side lengths (4.3 ft and 7.6 ft in Fig 3A) may tend to force the straight-line fit closer to the origin. This range effect may reveal nonlinearity in the relationship between room size and absolute error not captured by the LME model.

Second, because of the unbalanced design (see Methods), the ranges of room sizes varied across visual conditions. This variation may have contributed to a significant interaction between visual condition and room dimension. We examined potential interactions between these two variables by performing an ANOVA (see Method) with absolute error as the dependent variable. There was a significant interaction for the Non-Door Side estimates (F(5,322) = 7.422, p < .001), but not for the Door Side estimates (F(5,322) = 0.849, p = .516).

The interaction appears to have been due to two factors. The Mild Blur condition was tested over a narrow range of Non-Door Side lengths (16.6 ft to 23.7 ft). The nonmonotonic dependence of absolute error on length across this narrow range produced a linear fit with negative slope, unlike the positive slopes for the Control and other visual conditions. Second, the Severe Blur condition yielded a substantially steeper slope than the Control condition.

#### Spatial updating

After walking along the three-segment paths, subjects estimated their Distance and Direction to the Starting Location, and Distance and Direction to the beanbag Target. Performance was scored as Absolute Error in distance (the unsigned difference between the subject’s distance estimate and the physical distance) and Absolute Error in direction (the unsigned difference between the subject’s angular direction estimate and the physical angle, taking into account errors in the indicated quadrant when necessary.)

For practical reasons, the Control data for the four updating measures were obtained in one room, so we have no direct evidence on the potential effect of room size. But, since subjects could view the starting location and target directly in the Control condition, it is probable that room size would have little or no effect. For the other five visual conditions, the four updating measures were obtained in different spaces, according to our counterbalancing procedure.

An LME analysis including these five conditions showed that there were significant (p < .05) but weak effects of room area on distance estimates to the Start Location and Target Location, but not on the direction estimates. The effect of room size on the distance estimates produced a difference of only 1.06 ft in the mean absolute errors over the full range of room sizes. To simplify the presentation of results, we have therefore excluded room size as a variable. The subsequent results are based on data analysis without reliance on LME models and without inclusion of room-size effects.

**Fig 4:**
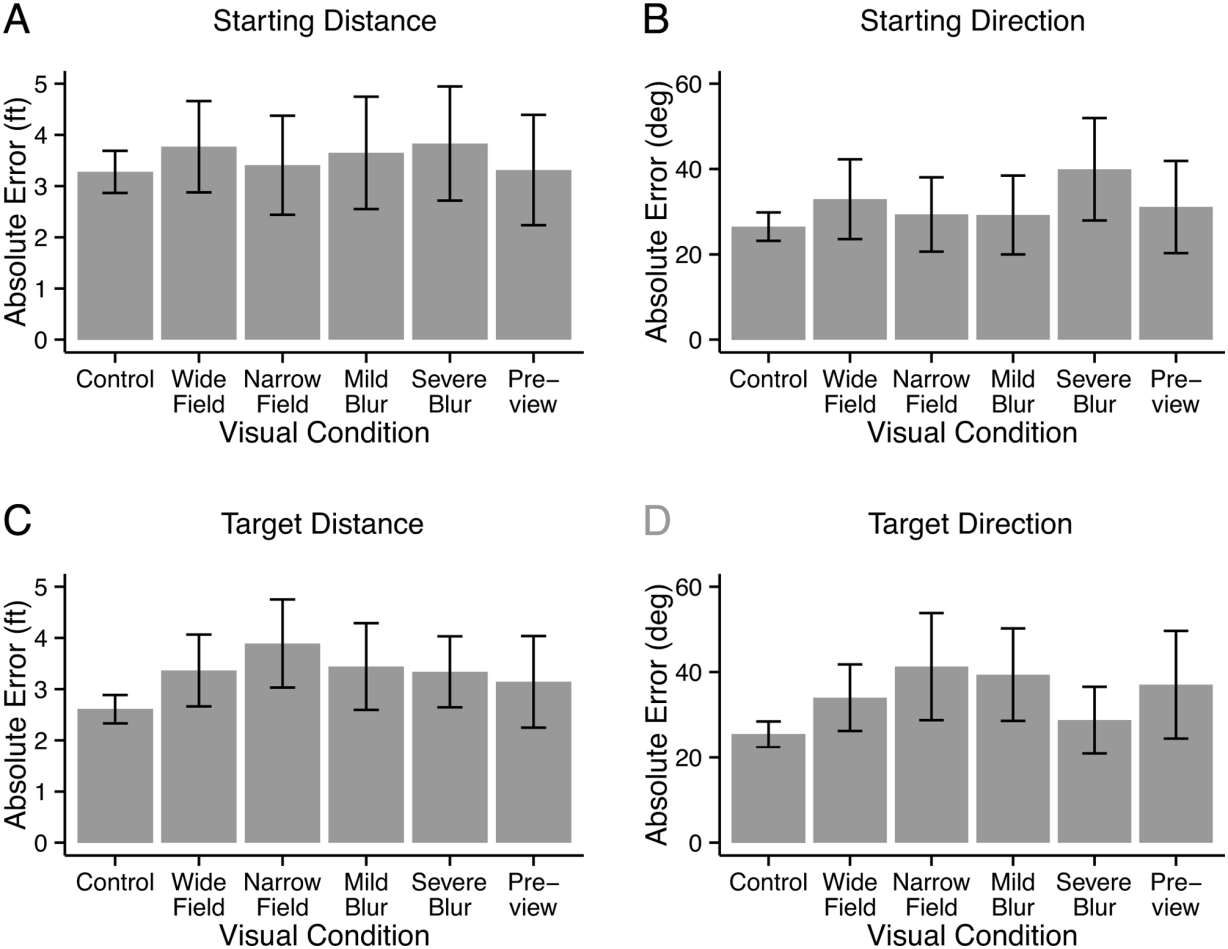
Experiment 1: Walking. Spatial Updating. Panels A and B show the mean absolute errors for estimates of the distance and direction to the Start Location (room door). Panels C and D show the mean absolute errors for estimates of the distance and direction to the Target Location (beanbag). Error bars represent 95% confidence intervals.

Fig 4 shows subjects’ mean absolute errors and confidence intervals. Distance and direction estimates to the Start Location (room door) are shown in Panels A and B, and corresponding results for distance and direction to the Target (beanbag) are shown in Panels C and D. The absolute errors are plotted for all viewing conditions including the Control Condition. Note that this differs from Panels C and D in Fig 3 where differences from the Control Condition, estimated from the LME model, were plotted.

For the Starting Distance, the mean Absolute Error in the Control condition was 3.27 ft. The average physical distance to the starting location in the control trials was 14.74 ft. The corresponding ratio (Weber fraction = mean absolute error / mean physical distance) was 0.22, close to the Weber fractions for estimating the room dimensions. Performance in estimating the Starting Distance (Panel A) was not affected by the viewing condition; all of the values were close to the Control value of 3.27 ft.

For the Starting Direction, the mean absolute error in the Control condition was 26.5°. The only visual-restriction that yielded poorer performance was Severe Blur with mean absolute error of 39.9°.

The results for the beanbag Target (panels C and D in Fig 4) differed from the results for the Starting Location in an interesting way; the visual-restriction conditions generally exhibited somewhat poorer performance (larger errors) than the Control condition. This was true for both distance and direction estimates, and did not seem to be specific to the type of visual restriction.

### Experiment 2: Wheelchair

In this experiment, the 16 Subjects were pushed along the three-segment paths in a wheelchair rather than walking. They made the same room dimension estimates for the lengths of the Door Side and Non-Door Side. They also estimated the distance and direction to the Starting Location, but the Target (beanbag) manipulation was omitted.

#### Room dimensions

As in Exp. 1, we computed mean ratios of subjects’ estimated length to true length for the five test spaces in Exp. 2, and also the overall mean ratios. For the Control condition, these ratios were: Door Side mean 0.90, range 0.85 to 0.97; Non-Door Side mean 0.95, range 0.93 to 0.98. These values are slightly below 1.0, indicating underestimates. For the Non-Door Side, the overall mean of 0.95 is close to the mean of 0.99 for the walking subjects, but significantly different according to an independent two-sample t-test (t = 3.18, p = .002). For the Door side, the mean ratio of 0.90 for the wheelchair subjects was quite different from the mean ratio of the walking subjects of 1.08 (t = 4.33, p = .00002).

**Fig 5:**
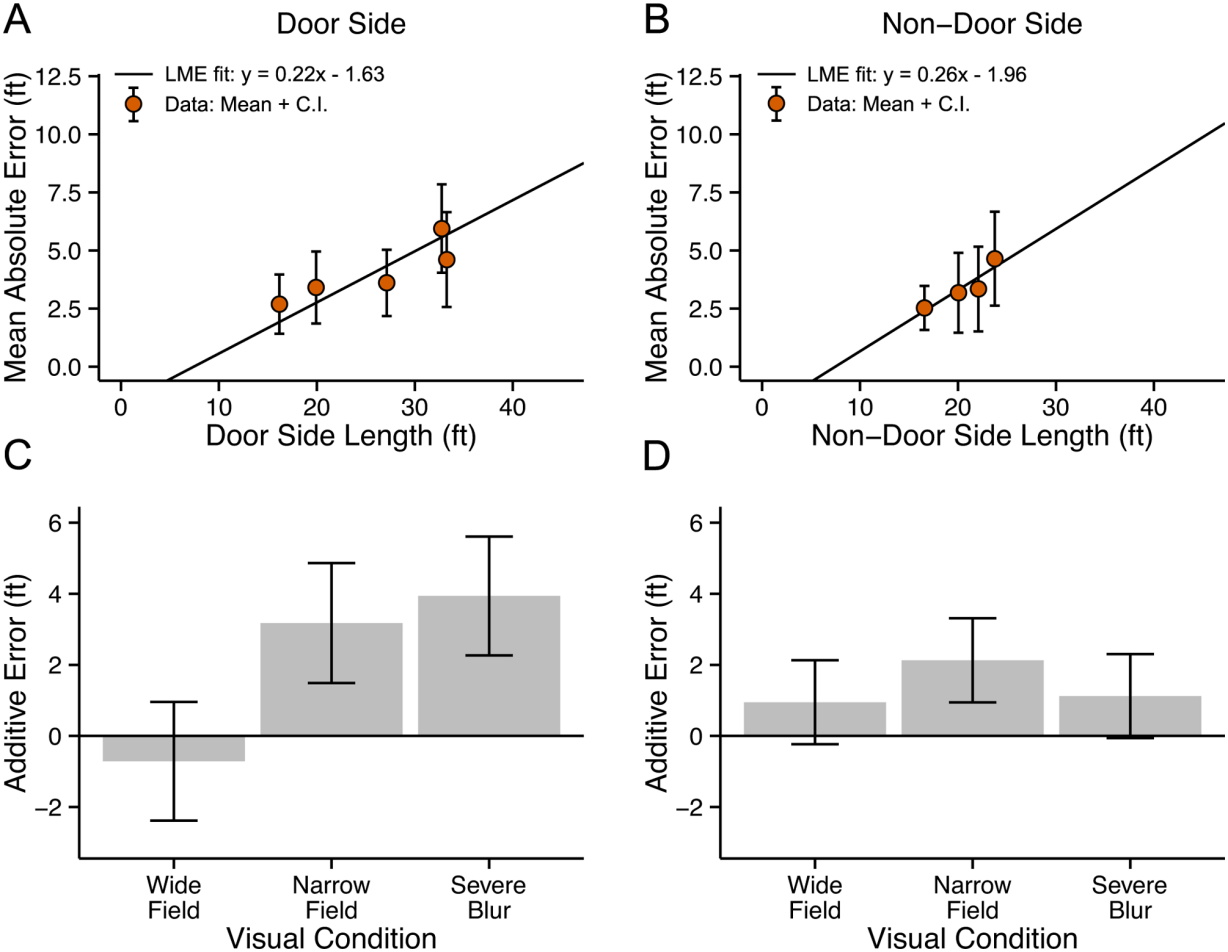
Experiment 2: Wheelchair. Estimates of Room Dimensions. Mean values and 95% confidence intervals for the absolute error in the Control condition are plotted as a function of physical length for the Door Side (panel A) and Non-Door Side (Panel B). Straight lines show the LME fit. Panels C and D show the additive errors associated with the three viewing conditions, estimated from the LME model.

In Fig 5, panels A and B plot mean absolute errors and their 95% confidence intervals as a function of the measured physical lengths of the Door Side and Non-Door Side of the five test spaces for control data. The straight-line fits are from the LME model with the relevant room dimension as a fixed effect and viewing condition as a fixed effect. These data are qualitatively similar to the walking data. The rising line indicates that absolute error increased as physical length increased. The slopes are similar for walking and wheelchair subjects. As noted in connection with Fig 3B, the negative values of y-axis intercepts imply negative absolute errors which are not possible. For reasons mentioned in the Methods, the two smallest rooms were omitted in Exp. 2. As discussed above in Exp. 1, lack of data for the smallest room dimensions, and some nonlinearity in the relationship between absolute error and physical length, may account for the negative intercepts.

Once again, we computed Weber fractions for room size estimation (the ratio of absolute error to physical length). For the Door Side, the overall mean Weber fraction for the wheelchair subjects was 0.20 (range across rooms 0.18 to 0.22), which is lower than the overall value of 0.26 for the walking subjects. For the Non-Door Side, the mean Weber fraction was 0.20 (range 0.15 to 0.25), which is almost identical to the mean value of 0.21 for the walking subjects.

Panels C and D in Fig 5 show the additional absolute errors associated with the other three visual conditions, relative to the Control condition (reference condition), based on the LME model. For the Door Side estimates (Panel C), these additive error estimates are substantially greater for both the Severe Blur (3.94 ft, CI = 1.67 ft) and Narrow Field (3.18 ft, CI = 1.69 ft). For the walking subjects, additive error for Severe Blur was also substantial (4.46 ft) but absolute errors for the Narrow Field condition did not differ from Control values.

For the Non-Door Side, the additive errors associated with the Narrow Field (2.13 ft, CI = 1.18 ft), and Severe Blur (1.12 ft, CI = 1.18 ft) were fairly similar to the values for the walking subjects for the Narrow Field (2.19 ft) and Severe Blur (2.81 ft).

Despite some quantitative variations, the overall pattern of results for the wheelchair subjects was similar to the results for the walking subjects. It appears that subjects in wheelchairs and walking subjects make similar room size estimates, with performance being notably poorer in conditions with narrow fields or severe blur.

#### Spatial updating

**Fig 6:**
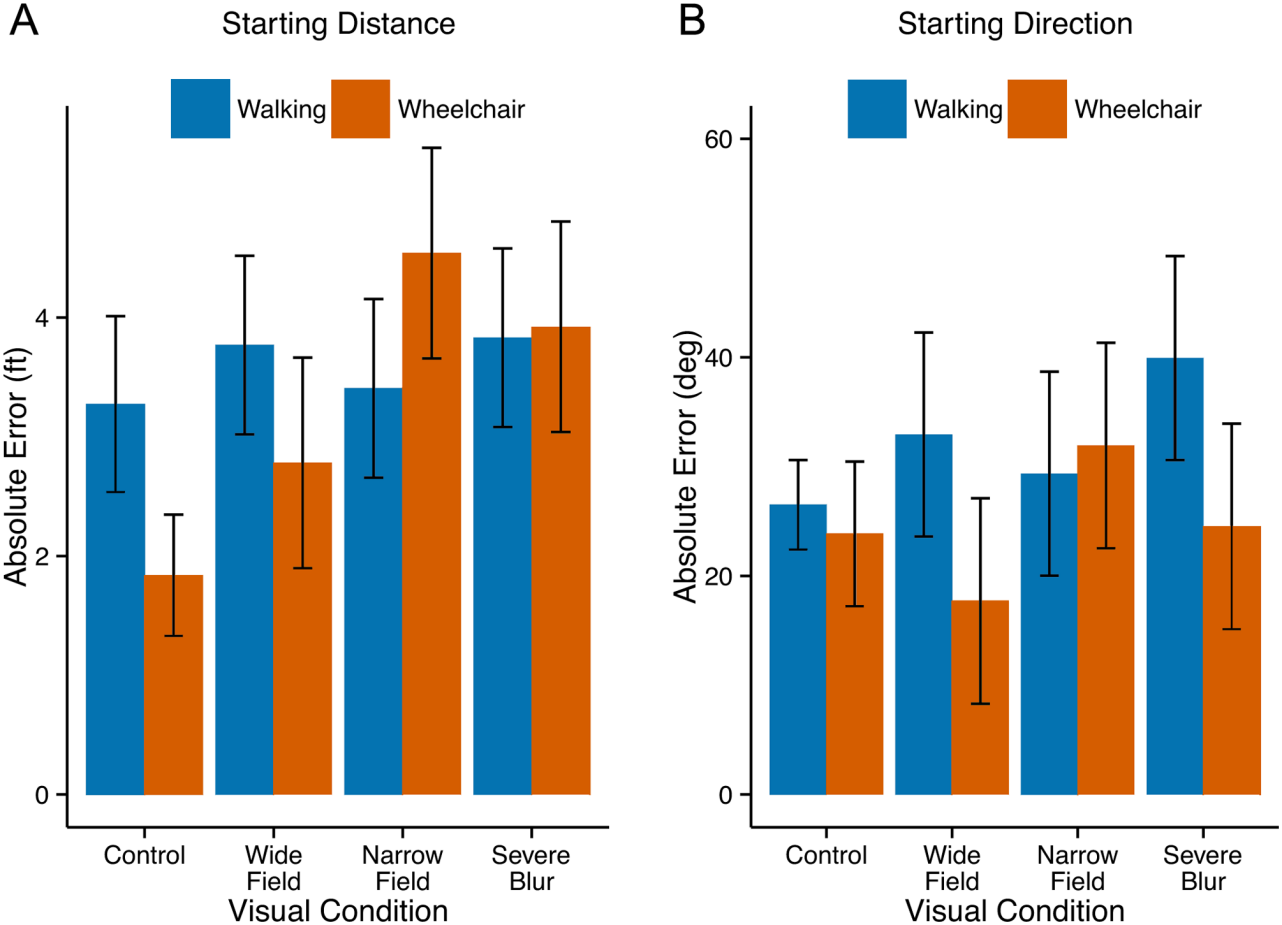
Experiment 2: Wheelchair. Spatial Updating. Panels A and B show the mean absolute errors for estimates of the distance and direction to the Start Location (room door). The orange bars are data for wheelchair subjects, and the blue bars are data for walking subjects replotted from Experiment 1. Error bars represent 95% confidence intervals.

After being pushed along the three-segment paths in the wheelchair, subjects estimated their Distance and Direction to the Starting Location. Once again, performance was scored as absolute error in distance and absolute error in direction. Fig 6 shows mean values and confidence intervals. The orange bars show the wheelchair results, and the blue bars replot the corresponding walking results from Fig 4 for comparison.

The key question here is whether being pushed in a wheelchair leads to poorer spatial updating, due to reduced proprioceptive cues. The answer appears to be negative. For the Control Condition, the wheelchair subjects actually have smaller absolute errors than the walking subjects in their distance estimates (panel A: Welch Two-Sample t-test, t(43.812) = 3.30, p < .01). For the Control condition, there was no significant difference between the two groups in direction estimates (panel B). Considering all eight comparisons between the two groups in Fig 6, the wheelchair group exhibited larger errors in only three of the comparisons. We conclude that there is no evidence that the wheelchair subjects make larger spatial-updating errors than the walking subjects.

The viewing condition had a significant effect on distance judgments for the wheelchair group (within-subject one-way ANOVA: F(3, 42) = 11.9, p < .001). This effect was likely due to the low absolute error in the Control condition (1.84 ft). Paired t-tests between the Control condition and the other conditions with adjustment for multiple comparisons, revealed a significant difference between the Control condition and the Severe Blur and Narrow-Field conditions, but not the Wide-Field condition. The viewing condition did not have a significant effect on the direction judgments for the wheelchair subjects.

## Discussion

### Room Dimensions

How well do normally sighted subjects estimate room dimensions? In the Control condition, the subjects had no viewing restrictions. In both experiments, the mean ratio of estimated length to true physical length was close to 1.0 for the Non-Door Side (0.99 in Exp. 1 and 0.95 in Exp. 2). A mean ratio of 1.0 implies no overall scaling error in the estimates, and confirms that the subjects were well calibrated in their verbal size estimates. The corresponding mean Weber fractions (ratio of average absolute error to true length) were close to 0.2 for both walking and wheelchair subjects, meaning an average error of 20% in the estimation of the Non-Door dimension. The corresponding results for the Door-Side dimension were a little more variable with the walking subjects in Exp. 1 overestimating length (average ratio 1.08) and the wheelchair subjects in Exp. 2 underestimating length (average ratio 0.90). The corresponding Weber fractions were close to 20%.

In the Control condition, the subject made room-size estimates while standing at the room doorway. The Non-Door Side estimate is an egocentric estimate of distance from oneself to an opposite wall. The Door-Side estimate is an exocentric estimate of the separation between walls to the left and right (although potentially construed as the sum of two egocentric judgments, one from the self to the right wall and the other from the self to the left wall). Several previous studies have compared egocentric and exocentric estimates of distance for localized objects on the ground plane. For a succinct review, see Geuss et al. [29]. Typically, Depth intervals along the line of sight are judged to be shorter than distance intervals in the frontal plane when a relative judgment is used, but when absolute measures are used (e.g., when subjects are asked to walk a distance blind-folded to match the observed distance), this difference disappears [30]. Geuss et al. [29] used a blind-walking task to directly compare exocentric and egocentric distances in real and virtual environments. In the real environments, subjects were quite accurate in both cases and showed no difference in the mean ratios of estimated distance to physical distance (both mean ratios were 1.03). Although our exocentric room-size estimates were slightly more variable than the egocentric estimates, our findings are generally consistent with the prior literature on absolute distance estimates for objects on the ground plane; subjects show similar accuracy in their judgements of egocentric and exocentric distances.

How did blur and field restriction affect room-size judgments? The results in Figs 3 and 5 compare the errors in these conditions with the control data. In both experiments, Severe Blur (Snellen equivalent of 20/900), but not Mild Blur (Snellen equivalent of 20/135), yielded larger errors in room-size judgments. The added error was greater for the Door-Side judgments than the Non-Door Side judgments. The Narrow Field (8°) was associated with greater error than controls (except in the Door-Side judgments in Exp. 1), with the errors generally being less than for the Severe Blur condition. The largest effect of visual restriction across all visual conditions was for the Door-Side judgments with Severe Blur in Exp. 1. Taken as a whole, Mild Blur did not affect room size judgments, the Narrow Field was associated with modest but measurable additional error, and Severe Blur produced more substantial errors. This was true whether subjects walked or were pushed in a wheelchair.

What cues might be used for estimating room size under conditions of visual restriction? Tarampi et al. [6] have shown that monocular estimates of egocentric distance to large objects at distances of several meters remain quite accurate for moderately severe acuity reduction (equivalent to Snellen 20/640). They argued that monocular cues for absolute distance on the scale of a few meters include target familiar size and the angle of declination between the subject’s straight-ahead line of sight and the target. While some of the walls in our test spaces may have had familiar objects near them (e.g., clocks, adjacent chairs, etc), we doubt that familiar-size cues were reliably available, especially under conditions of blur. A more reliable cue was likely the angle of declination between the line of sight and the wall-floor boundary. This boundary always had a large angular extent (the length or width of the room), helpful in offsetting acuity reduction. In most of the spaces, the contrast between the wall and the floor was quite high due to differences in surface coverings (e.g., carpeted floors and painted walls) or to differences in illumination from windows or overhead lights. Gibson [31] proposed that visible targets in the lower visual field are assumed to be on the ground plane, absent information to the contrary. In our case, it is reasonable to expect that subjects would assume a visible wall-floor boundary to be on the ground plane, and use the angle of declination to estimate distance to the wall. The good performance of our subjects in the Mild Blur condition is consistent with this possibility and the findings of Tarampi et al. [6]. The poorer performance in the Severe Blur condition may have been due to reduced visibility of the wall-floor boundary in some cases.

In the Narrow-Field condition (8° field of view), the wall-floor boundary would be out of the field of vision when the gaze direction was straight ahead. The subject would need to tilt gaze downward to search for the boundary, possibly adding noise to the angle of declination estimate. This may account for the slightly poorer performance in room estimation for the Narrow-Field condition. A related argument holds that severely restricted fields hamper encoding of spatial relations between features in spatial layouts by preventing direct eye movements between the features [32]. It has also been shown that severe field restriction can reduce accuracy of absolute distance judgments by occluding access to ground-plane cues, such as texture gradients, between the observer and the target [33].

Before discussing the spatial-updating results, we briefly consider whether our subjects treated the lengths and widths of rooms as independent judgments, as would be expected from the foregoing discussion of cues. We performed an LME analysis (see Methods) with these two judgments as dependent variables. In each case, Door-Side and Non-Door Side lengths and visual conditions were fixed effects. We also considered whether the judgments depended on the interaction between Door-Side length and Non-Door Side length. If the judgments were independent of one another, we would expect to find that judgments of Door-Side length would depend only on actual Door-Side length, and not on Non-Door Side length or the interaction of the two lengths. We would have corresponding expectations for the Non-Door Side judgments. In 11 of the 12 possible tests of significance (two types of judgments each for walking and wheelchair subjects, and three possible effects including interactions), the expectations based on independent judgments were confirmed. The one exception was a significant effect of the Door-Side length on judgments of the Non-Door Side length for the walking subjects. Apart from this exceptional case, the evidence indicates that the subjects treated length and width of the rooms independently.

### Spatial Updating

Spatial updating is the ability to keep track of current position and heading relative to a starting location during travel through a space. Spatial updating can be achieved by two general mechanisms [34]—either reference to stable landmarks in the environment (piloting), or use of cues to translations and rotations gathered during movement (path integration). In principle, path integration can use visual optic flow, afferent signals from joints and efferent signals to muscles (proprioception), vestibular signals concerning head accelerations, and also sensed duration of travel. There is evidence that cues from these different sources combine to create a single multimodal representation of movement through a spatial layout [35].

In Exp. 1, subjects walked a three-segment path and then estimated the distance and direction to the starting location, and also to a target (beanbag) dropped at the first waypoint. In the control conditions, the subjects were allowed to freely view the starting location and target so that their estimates could rely on direct visual observation. In the remaining visual conditions, The subjects were not allowed to look back at the starting location or target, so their estimates had to rely on other spatial-updating cues.

There was no effect of visual condition on the ability to estimate distance back to the starting location. Even in the Preview condition, in which subjects were blindfolded during the walk, errors in the distance estimates did not differ significantly from the control values. With the exception of the Severe Blur condition, errors in the direction estimates to the starting location were similar for the control condition and the other visual conditions.

If our subjects relied on vision of landmarks in the room or on optic flow for spatial updating, we would expect to find an effect of the visual-restriction conditions. The lack of dependence on the nature of the visual input implies that non-visual body-centered cues were sufficient for our walking subjects in Exp. 1.

These results are consistent with previous findings showing that visual cues are not necessary for spatial updating. Although optic flow can be used to estimate distance travelled [36], several studies have shown that pure optic flow results in poor spatial updating, especially when turns are present [11, 37, 38]. Although visual cues may not be necessary for spatial updating tasks such as ours, it is possible that previous visual experience is required to calibrate proprioceptive or vestibular cues in path integration [39].

Rand, Creem-Regehr and Thompson [9] found that moderately severe blur resulted in poorer memory for the location of several targets seen by subjects while navigating through corridors in a building. They provided evidence that the performance deficit was due to extra attentional demands from navigating with degraded vision. In our experiments, the attentional demand was low; subjects were guided along simple paths and had to recall only one or two locations.

In Exp. 2, Subjects made their judgments while seated in a wheelchair. Their ratios of room-size estimates to true physical lengths were below 1.0, and lower than those for walking subjects. While we can’t be sure of the reason for the difference between the two groups, one possible factor is that the wheelchair subjects viewed the rooms with a lower eye height than the walking subjects. It is known that the angle of declination (angle between an observer’s line of sight parallel to the ground plane and a target on the ground) can influence estimates of absolute egocentric distance to a target [40]: the smaller this angle, the farther the target. Reducing eye height reduces the angle of declination and, if there is no recalibration, would make a target seem farther away. A possible cue to the distance of a wall in a room is the angle of declination between gaze direction and the boundary between the floor and the wall; this angle would be smaller for a more distant wall. If our seated subjects used a default angle of declination based on eye height from standing, their distance estimates would increase. This is the opposite of what we observed. This means that if our wheelchair subjects used the angle of declination cue, they must certainly have compensated in some way for their reduced eye height. A speculative possibility is that they tilted their gaze upward while providing verbal responses to the standing experimenter, thereby increasing the angle of declination. We have no direct evidence that our subjects used the angle of declination cue in our experiments. But Rand et al. [41] have shown that normally sighted subjects with artificial blur do make use of a floor-wall boundary (“visible horizon” in their terminology) in judging the distance to objects on the floor.

Our primary goal in Exp. 2 was to limit proprioceptive cues by pushing subjects along the travel paths in a wheelchair to see if they would exhibit poorer spatial-updating performance than the walking subjects in Exp. 1. We also reasoned that reduction of proprioceptive input might force greater reliance on visual cues and therefore reveal a greater dependence on visual condition. Contrary to these possibilities, the wheelchair subjects did not exhibit poorer updating performance than the walking subjects, nor did they show more dependence on visual condition. The reduced proprioceptive input did not have an adverse impact on spatial updating for our wheelchair subjects.

The robustness of spatial-updating performance in the presence of degraded visual and proprioceptive input implies an important role for vestibular cues in our spatial-updating task. Previous studies have shown the importance of vestibular cues in spatial updating. Klatzky, Loomis et al. [38] demonstrated the necessary role of vestibular cues in coding rotations in path integration by comparing updating performance with cues from optic flow alone, or from actual walking, or from optic flow plus rotations in a swivel chair. Arthur et al. [42] found that subjects with unilateral vestibular hypofunction underestimated distances traversed in guided blind walking. Allen et al. [43] compared the performance of young (mean age 20.7) and old (mean age 72.3) subjects in a triangle completion task. Subjects were blindfolded and were either led along a two-segment path or pushed in a wheelchair. For the younger subjects, there was very little difference in the two conditions, but the older subjects exhibited poorer performance in the wheelchair condition. The results imply an age-related decline in the usefulness of vestibular cues for spatial updating. This finding may be pertinent to low vision because of the greater prevalence of low vision in the older population.

Vestibular cues provide information about acceleration and not distance per se. It’s possible they play a dominant role in spatial updating for short paths with multiple starts, stops and turns such as ours. Siegle, Campos et al. [44] showed that the reliability of vestibular cues for path integration may decline for more prolonged translational movements at constant velocity.

Mittelstaedt and Mittelstaedt [45] presented evidence that either vestibular or proprioceptive cues are sufficient for path length estimation. They argue that when both types of cues are present, such as in blindfolded walking, path integration is primarily reliant on proprioceptive cues. It is possible that our wheelchair subjects used vestibular cues for path integration while our walking subjects relied more on proprioception.

Taken together, our findings and those in the literature imply that spatial updating is not much affected by artificially reduced acuity or narrow field of view. Apparently, subjects can keep track of their current position and orientation relative to a starting location using proprioceptive and/or vestibular cues.

Our subjects’ estimates of the distance and direction to the beanbag target in Exp. 1 differed in an important way from their estimates to the starting location. Unlike results for the starting location, performance in the various visual conditions was generally poorer than in the control condition. Why would subjects have equivalent accuracy for locating the Starting Location and the Target in the Control condition, but poorer accuracy for locating the Target in the visual-restriction conditions? In the Control condition, subjects could look back at the Target or Starting locations before making their distance and direction estimates. In the visual-restriction conditions, subjects were not allowed to look back, so their estimates needed to rely on internal spatial coding of position. Greater errors for Target localization may reflect less precise spatial coding than for the Starting location. The less precise coding of target position might be due to less time spent in the vicinity of the target (it was simply a waypoint on the three-segment path) or to the arbitrariness of the location of the target in the room.

A peculiarity of the Target Direction data is that the absolute error for Mild Blur (39.4°) was substantially larger than for Severe Blur (28.7°). This would seem to indicate that the poorer performance is not due to the extent of visual restriction. This is consistent with the possibility that the encoding of target location is less precise overall than encoding of starting location.

Finally, we comment briefly on the Preview condition in Exp. 1. In this condition, subjects were given 10 seconds to view the space from the doorway without any visual restriction. They were then blindfolded, guided along the three-segment path and then made their room-dimension and spatial-updating responses. Performance on this condition did not differ from control performance on any of the measures. We included this condition primarily for comparison with two entirely nonvisual conditions, not described in detail in this paper. One of these conditions involved walking the three-segment path blindfolded and with auditory masking, that is, no visual or auditory cues. Prior research has shown that visual preview can sometimes facilitate spatial updating in a blind-walking task. In our case, however, spatial updating performance did not differ between blind-walking conditions with and without preview. Philbeck, Klatzky et al. [16] provided evidence that preview helps spatial updating in a blind walking task if landmarks are visible along the path to be taken, but not helpful if the landmarks are remote from the path. In our case, the major landmarks in the rooms—furniture, windows, wall features—were not associated with the path. There were unobtrusive tape marks on the floor defining the path waypoints. Our informal impression is that the subjects did not pay attention to the tape markings. If we are right, then our lack of a preview benefit in spatial updating is consistent with Philbek, Klatzky et al.’s [16] finding.

### Implications for Low Vision and Blind Pedestrians

Our study was motivated by an interest in the impact of impaired vision on access to global features of indoor environments. This interests stems from our program of research on designing visually accessible spaces. A pedestrian’s safe and effective mobility in indoor spaces can benefit from knowledge of the size and shape of layouts, and also the ability to keep track of one’s position and orientation in the space.

In the present study, we have examined the ability of normally sighted subjects to make spatial- updating and room-size judgments with artificial restrictions of acuity and visual field. These measurements have provided insight into the role of vision in providing information for these functionally relevant tasks, and also baseline information for comparison with the performance of blind and low-vision subjects.

We found that severe blur led to larger errors in room-size judgments. Assessment of room size may depend on detection and utilization of surface features on walls or the bounding contour between floors and walls. The walls in our test spaces were often fairly homogeneous, containing only fine texture and occasional larger objects such as windows and pictures. Fine texture is not visible with severe blur or low acuity, so distance judgments to wall surfaces would need to rely on the larger features, whose distance might be difficult to judge because of uncertain size and height in the field of view. For people with very low acuity, high-contrast boundaries between floors and walls, and large wall features of known size and position might facilitate room size judgments. The narrow-field condition also resulted in greater room-size errors, perhaps due to difficulty in using texture gradients as a cue to distance or in locating the bounding contours between floors and walls.

The lack of visual effects on the spatial-updating measures would lead us to expect that visually-impaired subjects would not differ from sighted subjects in spatial updating. But in real-world contexts, the paths taken by people are often much more complex than the three-segment paths studied here, and pedestrians may be distracted by other activities in the space. Imagine, for example, circulating at a party or reception, moving from place to place and engaging in conversation, while trying to keep track of one’s position and orientation relative to the entrance to the space. As the number of path segments increases, path-integration errors in position and orientation would increase, if no method of error correction is possible. A person with normal vision can use familiar landmarks in the space for error correction. To the extent that such landmarks are not visible for a person with low vision, difficulties in spatial updating may occur. Large, highly visible features of known size and location in the space could prove useful as landmarks for people with low vision.

In a subsequent paper, we will compare the performance of blind and low-vision subjects with the performance of normally sighted subjects described in this paper. We will also evaluate the use of ambient auditory cues by normally sighted, blind and low-vision subjects in room-size and spatial-updating judgments.
